# Expression Characteristics of microRNA in Pig Umbilical Venous Blood and Umbilical Arterial Blood

**DOI:** 10.3390/ani11061563

**Published:** 2021-05-27

**Authors:** Mailin Gan, Lin Liu, Shunhua Zhang, Zongyi Guo, Ya Tan, Jia Luo, Qiong Yang, Hongmei Pan, Xuewei Li, Jinyong Wang, Linyuan Shen, Li Zhu

**Affiliations:** 1 College of Animal Science and Technology, Sichuan Agricultural University, Chengdu 611130, China; ganmailin@stu.sicau.edu.cn (M.G.); 2018302012@stu.sicau.edu.cn (L.L.); 14081@sicau.edu.cn (S.Z.); tanya@stu.sicau.edu.cn (Y.T.); xuewei.li@sicau.edu.cn (X.L.); 2 Farm Animal Genetic Resource Exploration and Innovation Key Laboratory of Sichuan Province, Sichuan Agricultural University, Chengdu 611130, China; 3 Chongqing Academy of Animal Science, Rongchang, Chongqing 402460, China; guozy@cqaa.cn (Z.G.); 2002019@swu.edu.cn (H.P.); kingyou@vip.sina.com (J.W.); 4 College of Animal Science and Technology, Southwest University, Chongqing 400715, China; b20161705@swu.edu.cn; 5 Department of Animal Husbandry and Veterinary Medicine, Chengdu Agricultural College, Chengdu 611100, China; 2018302019@stu.sicau.edu.cn

**Keywords:** pig, umbilical cord blood, miRNAs, diseases

## Abstract

**Simple Summary:**

Umbilical cord blood is an important biological resource for research on fetal development and treatment of related diseases. To further explore the biological functions of umbilical cord blood, we sequenced the small RNAs in porcine umbilical venous blood and umbilical arterial blood and analyzed them through two differential expression analysis methods. Through comparative analysis, it is found that highly expressed miRNAs in umbilical venous blood and umbilical artery blood perform similar functions, but there may be differences in immunity and angiogenesis. Because miRNAs are highly conserved and have the ability to transmit across species, they have great potential in the treatment of human-related diseases. Further analysis shows that cord venous blood miRNA may be helpful in the treatment of muscle-related diseases, while cord arterial blood miRNA may be beneficial in the treatment of liver- and brain-related diseases. This research helps to analyze the biological functions of cord blood miRNA and provides a reference for the development and application of porcine cord blood miRNA-related products. At the same time, it provides new insights for the further development of value-added products in the animal husbandry industry and the treatment of human diseases.

**Abstract:**

As the medium of material exchange between mother and fetus, umbilical cord blood is closely connected with fetal development. microRNA (miRNA) has a wide range of biological functions and has high flow characteristics. Small RNA sequencing of pig umbilical venous blood (UVB) and umbilical arterial blood (UAB) revealed that a total of 302 miRNAs were identified, and 106 and 22 miRNAs were specifically expressed in the UVB and UAB, respectively. Using the two methods of differential expression multiple and differential expression percentage, it is found that only 35% of the highly expressed miRNAs in the UVB by the two analysis modes overlap, but 56.25% of the enriched signal pathways are the same. Only 20% of the highly expressed miRNAs in the UAB overlap, but 62.07% of the signal pathways are the same. Further analysis revealed that miR-423 can be used as a characteristic miRNA of UVB and has the potential to treat muscle-related diseases. miR-122-5p can be used as a characteristic miRNA of UAB and may help to improve liver- and brain-related diseases. In summary, these results enrich understanding of miRNA in mother–fetal communication and provide a reference for the development and application of porcine cord blood products.

## 1. Introduction

There are usually one umbilical vein (UV) and two umbilical arteries (UAs) in the umbilical cord (UC). Umbilical vein blood (UVB) delivers oxygen and nutrients to the fetus, and umbilical arterial blood (UAB) takes away the carbon dioxide and metabolic waste produced by the fetus [1]. Cord blood is not only rich in nutrients, but it is also rich in hematopoietic stem cells, which can be used for hematopoietic stem cell transplantation and treatment of diseases of the blood system and the immune system [2]. Therefore, cord blood has become an important biological resource [3].

microRNA (miRNA) is a type of endogenous small RNA with a length of approximately 20–24 nucleotides, which has a variety of important regulatory effects in the organism [4]. It is speculated that miRNA regulates one-third of the gene expression in animals. There, it usually complements the 3′ UTR regions of the target gene through the seed sequence and inhibits gene expression at the post-transcriptional level. In recent years, there have also been reports about miRNA functioning by binding to the 5′ UTR [5] and CDS regions of genes [6]. Each miRNA can have multiple target genes, and several miRNAs can also regulate the same gene [7]. This complex regulatory network can not only regulate the expression of multiple genes through one miRNA but also finely regulate the expression of a certain gene through the combination of several miRNAs [8]. miRNAs of different species are not only conserved in gene position but also show a high degree of homology in sequence [9]. In addition, miRNA expression patterns in different tissues of different species are also similar. The high degree of conservation of miRNA is closely related to the importance of its function. Since the sequence of miRNA is also highly conserved among different species, miRNA has the potential to play an important role in cross-species regulation. Previous studies have found that plant miRNAs enter animals and play a role after being eaten by animals [10]. Therefore, it can be inferred that animal-derived miRNAs can also enter other animals’ bodies to regulate their physiological functions.

Pigs are widely raised large animals. There are billions of newborn pigs in the world every year, and pigs are highly similar to humans in cardiovascular aspects [11]. Therefore, pig umbilical cord blood-derived products have great potential in human medicine as an important renewable biological resource [12]. The development of cord blood miRNA will be one of the important directions. It is worth mentioning that pigs have multiple births. They have the advantages of collecting cord blood in a centralized and batch manner that other domestic animals do not have [13]. Therefore, to further understand the expression characteristics of pig cord blood miRNA, the miRNAs in umbilical cord blood arteries and veins were sequenced, and the differential expression of porcine cord blood miRNAs in arteries and veins was analyzed using two analysis methods [14] in order to provide a reference for the further development and utilization of porcine cord blood miRNAs.

## 2. Materials and Methods

### 2.1. Ethics Statement

All experiments were conducted in accordance with the requirements and standards of the Sichuan Agricultural University Ethics Committee (Sichuan, China, No. DKY-B20131403).

### 2.2. Animals and Treatment

Six boars (Yorkshire piglets) from six different sows (the fathers of all six piglets were also different) were obtained from Mianyang Mingxing Agricultural Technology Development Co., Ltd. (Mianyang, Sichuan, China). Three pig UVB and UAB plasma samples were mixed into one sample, and then small RNA sequencing was performed [15]. In addition, 10 healthy boars (Yorkshire piglets) born on the same day were used to measure the correlation between umbilical cord and birth weight. A 15% EDTA anticoagulant solution was prepared in advance, and 80 μL was added to the 5 mL syringe before each collection of umbilical cord blood. After the piglets were born, one person took care of them, and the other quickly found the umbilical vein and the umbilical artery under the warming lamp and inserted the marked syringe into the umbilical vein and the umbilical artery to draw blood. After the blood was drawn, it was quickly mixed upside down and transferred to a 1.5 mL centrifuge tube without RNase. In total, 0.5 mL blood was taken for routine blood testing, and the remaining blood samples were used for plasma separation. After the umbilical cord blood collection was completed, the staff took care of the piglets and sows and marked their ear numbers for follow-up observation. In addition, three sows with a litter of more than 14 were selected, and one piglet was killed in each litter for the analysis of tissue gene expression profiles. When collecting the internal organs of the piglets, 0.5 g samples were taken from three different parts of the same internal organs (the sampling parts of each piglet were the same) and quickly stored in liquid nitrogen. After the samples were transported back to the laboratory, the samples from three different parts of the same organ were ground and mixed thoroughly to form a visceral tissue sample.

### 2.3. Analysis of Sequencing Data

The process of small RNA sequence data processing and analysis is referred to in previous research [16]. First, remove low-quality reads and adapter sequences to obtain clean reads. Subsequently, use the Burrows–Wheeler alignment (BWA) mapping tool to map the clean data to the pig reference genome (Sscrofa10.2). Through BLAST search, unique sequences with a length of 18–26 nt are mapped to species-specific precursors in miRBase to identify known miRNAs and new 3p- and 5p-derived miRNAs [17]. Sequences containing different lengths at the 3′- and 5′-ends and single mismatches within the sequences are retained in the alignment. Species-specific mature miRNAs with unique sequences mapped to hairpin arms are identified as known miRNAs (GSE87111). The two analysis methods of differential expression multiple (up/down: |log2Fold change| ≥ 1.5) and differential expression percentage (lose/gain: |UV-UA| ≥ 0.2 (it was higher than 83% miRNAs in UVB and 71% miRNAs in UAB)) were used to analyze the expression characteristics of pig umbilical venous blood and umbilical artery blood. To avoid false positives, the real-time quantitative PCR (RT-qPCR) method is used to detect miRNAs where the two analysis methods overlap.

### 2.4. Target Prediction and Functional Annotation of Target Genes

Targetscan (http://www.targetscan.org/vert_72/, accessed on 15 June 2020) [18] and miRBD (http://mirdb.org/, accessed on 15 June 2020) [19] were used for target gene prediction (using human database as a reference), and DAVID (https://david.ncifcrf.gov/conversion.jsp?VFROM=NA, accessed on 19 June 2020) was used for GO (Gene Ontology) and KEGG (Kyoto Encyclopedia of Genes and Genomes) annotation of target genes [20].

### 2.5. Real-Time Quantitative PCR

Total RNA from the tissues was extracted using RNAiso Plus (TaKaRa, Dalian, China) and plasma RNA was extracted using RNAiso Blood (TaKaRa). After RNA extraction, NanoDrop ND-2000 (Thermo Scientific, Waltham, MA, USA) was used to detect RNA concentration (>200 ng/µL) and purity (1.8 < OD_260/280_ < 2.1). Denaturing agarose gel electrophoresis was used to detect RNA integrity (the brightness of 28S rRNA bands was nearly twice that of the bands of 18S rRNA). Reverse transcription of miRNAs was done according to the kit instructions (TaKaRa). qRT-PCR was performed using the SYBR Premix Ex Taq kit (TaKaRa) on a CFX96 RT-PCR detection system (Bio-Rad, Richmond, CA, USA). To calculate relative miRNA expression, the 2^−ΔΔ^Ct method [21] was used with U6 as the internal reference [15]. The primer sequences used for RT-qPCR are shown in Table S1.

### 2.6. Blood Routine Examination

The umbilical cord blood was collected in a test tube containing EDTA anticoagulant. The automatic blood cell analyzer BC-2800vet (Mindray, Shenzhen, China) was used to evaluate the type and number of cord blood cells.

### 2.7. Statistical Analysis

Analysis of differentially expressed miRNAs in the sequencing results was carried out using the R software package edgeR. All quantitative results are expressed as mean ± SEM. SPSS20.0 software was used for statistical analysis. Data analysis adopts one-way analysis of variance. The *t*-test and the multiple comparison test were used to analyze the differences between groups. If *p* < 0.05, the difference between the averages is considered statistically significant.

## 3. Results

### 3.1. Characteristics of Pig Umbilical Cord

**Figure 1:**
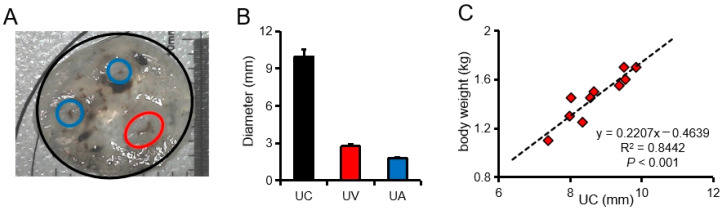
Characteristics of pig umbilical cord and its effect on birth weight. (**A**) Cross section of pig umbilical cord. (**B**) Diameters of pig umbilical cord (UC), umbilical vein (UV), and umbilical artery (UA). (**C**) Correlation analysis between pig umbilical cord diameter and newborn pig weight. *N* = 10.

Like most mammals, the umbilical cord of a pig fetus has one umbilical vein and two umbilical arteries (Figure 1A). Usually, the diameter of the umbilical vein is larger than that of the umbilical artery (Figure 1B). Here we also found that there is a significant positive correlation between the diameter of the umbilical cord of pigs and the birth weight of newborn piglets (Figure 1C). Further analysis of the UVB and UAB indicators revealed that there was no significant difference in the blood indicators (Table 1).

### 3.2. Expression Analysis of miRNAs in Pig UVB and UAB

**Figure 2:**
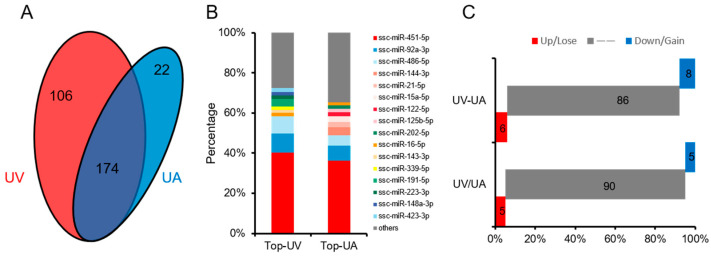
Characteristics of pig umbilical cord and its effect on birth weight. (**A**) The Venn diagrams represent all the numbers of expressed miRNAs. (**B**) Composition of highly expressed miRNAs. (**C**) Differential expression of the top 100 miRNAs in UVB and UAB (up/down: |log2Fold change| ≥ 1.5, lose/gain: |UV − UA| ≥ 0.2).

Using small RNA sequencing technology, analysis of UVB and UAB miRNAs revealed that UVB and UAB co-express 174 miRNAs, and 106 unique miRNAs were expressed in UVB and 22 miRNAs in UAB (Figure 2A). Analyzing highly expressed miRNAs in UVB and UAB, it was found that 4 of the top 10 miRNAs expressed in UVB and UAB were the same (Figure 2B). Utilizing differential multiple analysis, it was found that, compared with UAB, 11 miRNAs were up-regulated and 10 miRNAs were down-regulated in UVB (Figure 2C). Through differential percentage analysis, it was found that among the first 100 miRNAs, UVB lost 16 miRNAs and gained 20 miRNAs compared with UAB (Figure 2C).

### 3.3. Prediction of miRNA Target Genes, and Analysis of GO and KEGG Pathways

**Figure 3:**
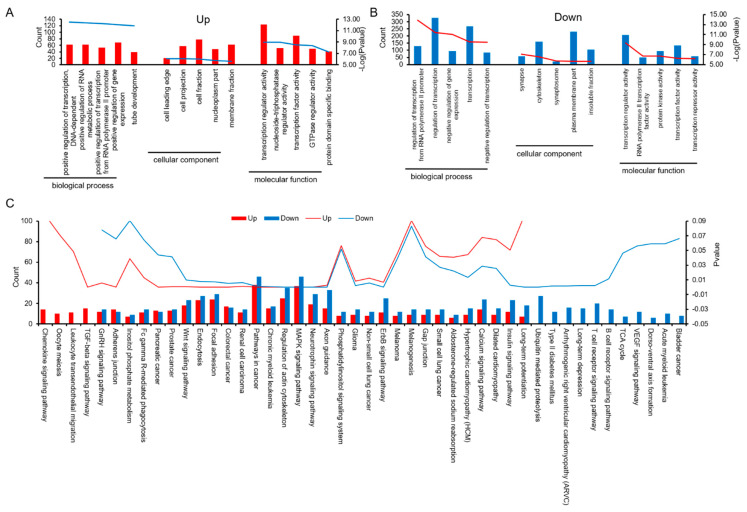
Prediction of miRNA target genes and analysis of GO and KEGG pathways based on expression difference percentage. (**A**) Gene ontology–biological process enrichment of target genes for up-regulated expressed miRNAs. (**B**) Gene ontology–biological process enrichment of target genes for down-regulated expressed miRNAs. (**C**) Functional enrichment analysis of differential miRNA in the normal pig UVB and UAB.

Prediction of miRNA target genes and analysis of GO and KEGG pathways based on expression difference multiples were carried out. The miRNA target gene prediction software was used to predict the target genes of up-regulated and down-regulated miRNAs, and GO enrichment analysis was performed on these speculative target genes. Interestingly, although the up-regulated miRNAs in cord venous blood are different from the down-regulated miRNAs, the GO analysis of their target genes has the following common features: ① In the analysis of biochemical processes, genes involved in “transcriptional regulation” are most enriched. ② Analysis of cellular components showed that “plasma membrane” genes were most enriched. ③ Analysis of molecular function showed that most genes were clustered into binding activities in both UVB and UAB (Figure 3A,B and Table S2). KEGG pathway annotations show that 67.39% of the signal pathways overlap in the target genes of up-regulated miRNA and down-regulated miRNA, which are mainly enriched in pathways in cancer, MAPK signaling pathway, regulation of actin cytoskeleton, focal adhesion, and neurotrophin signaling pathway. The target genes of up-regulated miRNAs are individually enriched in 8.70% of the signal pathways (TGF-beta signaling pathway, leukocyte transendothelial migration, oocyte meiosis, and chemokine signaling pathway). Down-regulated miRNA target genes alone enriched 23.91% of the signaling pathways, mainly in ubiquitin-mediated proteolysis, T cell receptor signaling pathway, and arrhythmogenic right ventricular cardiomyopathy (Figure 3C).

**Figure 4:**
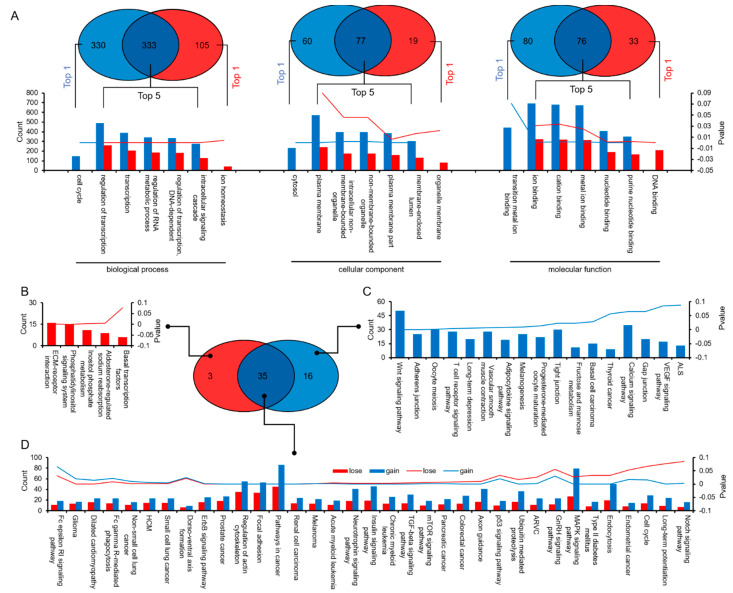
Prediction of miRNA target genes and analysis of GO and KEGG pathways based on expression difference percentage. (**A**) GO analysis of the target genes for lose/gain miRNAs. (**B**) KEGG annotation enrichment of target genes for lose/gain miRNAs.

Prediction of miRNA target genes and analysis of GO and KEGG pathways based on expression difference percentage were carried out. The miRNA target gene prediction software was used to predict the target genes of “lose” and “gain” miRNAs, and GO enrichment analysis was performed on these speculative target genes. The GO analysis based on the lose and gain miRNAs, respectively, found that the overlapping pathways in the biochemical processes accounted for 50.23% of the “lose” pathway and 76.03% of the “gain” pathway. In the cellular components, overlapping pathways account for 56.20% of the lose pathways and 80.21% of the gain pathways. In the molecular function, the coincident pathway accounts for 48.72% of the lose pathway and 69.72% of the gain pathway (Figure 4A and Table S2). KEGG pathway annotations show that 61.40% of the signal pathways overlap in the target genes of lose miRNA and gain miRNA, which are mainly enriched in pathways in cancer, regulation of actin cytoskeleton, focal adhesion, MAPK signaling pathway, and endocytosis (Figure 4B).

### 3.4. Similarities and Differences between the Two Differential Expression Analysis Modes

**Figure 5:**
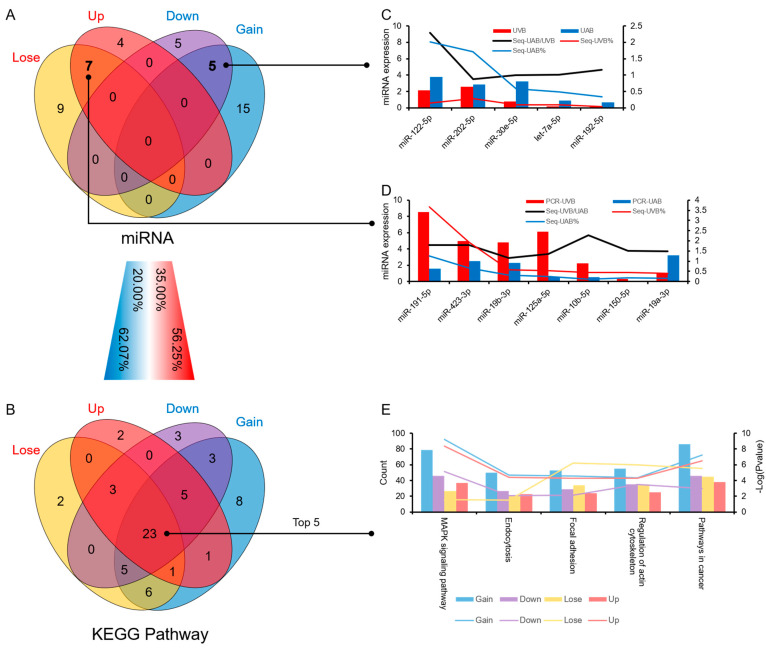
Comparison of the two differential expression analysis modes. (**A**) The Venn diagram shows the overlap between the differentially expressed miRNAs in the two comparison methods. (**B**) The Venn diagram shows the overlap between the differentially KEGG pathway in the two comparison methods. (**C**,**D**) The expression of miRNAs detected by RT-qPCR. C: “down” and “gain”; D: “up” and “lose.” *N* = 3. (**E**) The top five signal pathways that are collectively enriched.

Through the comparative analysis of the two methods, it was found that although only 35% of the high-expression miRNAs in UVB were coincident. The same signal pathway was enriched in 56.25%. Only 20% of the highly expressed miRNAs in UAB obtained by the two methods were the same, but 62.07% of the signaling pathways overlapped (Figure 5A,B). Using RT-qPCR to verify the overlapping miRNAs obtained by the two analytical methods, it was found that expression trends of overlapping miRNAs except for miR-19a-3p were similar to those of sequencing (Figure 5C,D). KEGG annotation analysis showed that the target genes of the four sets of miRNAs obtained by the two analytical methods collectively enriched 37.10% of the signal pathways (Figure 5E).

### 3.5. The Influence of Typical Differential miRNAs in Umbilical Cord Blood on Disease Occurrence and Its Potential Application Value

**Figure 6:**
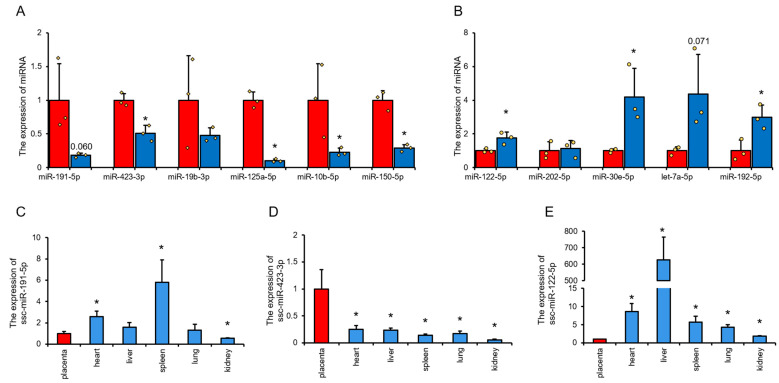
Tissue expression profile of characteristic miRNAs. (**A**) The expression level of up-regulated miRNA in cord venous blood was detected by RT-qPCR. (**B**) The expression level of down-regulated miRNA in cord venous blood was detected by RT-qPCR. (**C**–**E**) The expression of miR-191-5p (**A**), miR-423-3p (**B**) and miR-122-5p in placenta and piglet tissue (heart, liver, spleen, lung, and kidney). *N* = 3. * *p* < 0.05.

Using RT-qPCR to detect and analyze the differentially expressed miRNAs of UVB and UAB, it was found that miR-423-3p, miR-125a-5p, miR-10b-5p, and miR-150-5p were significantly higher in UVB than UAB; miR-122-5p, miR-30e-5p, and miR-192-5p were significantly lower in UVB than UAB (Figure 6A,B). These results indicate that the RT-qPCR results are highly consistent with the sequencing results. To understand the expression characteristics of UVB and UAB, differential miRNAs in various tissues and organs of the fetus were studied. Further quantitative detection of miRNAs with a difference of more than 2 and a change of more than 0.5% showed that miR-191-5p, miR-122-5p, and miR-423-3p are expressed in the placenta and neonatal pig tissues and organs. miR-191-5p is highest expressed in the spleen and lowest in the kidney of neonatal pigs (Figure 6C), miR-423-3p is highest in sow placenta and lowest in newborn pig kidney (Figure 6D), and miR-122-5p is highest in newborn pig liver and lowest in sow placenta (Figure 6E). Depending on the tissue expression pattern of miRNA, miR-423 and miR-122-5p can be used as characteristic miRNAs of UVB and UAB, respectively. By comparing with the Mammalian ncRNA-Disease Repository (NMDR), it is found that umbilical venous blood may have potential for the treatment of muscle-related diseases, while umbilical artery blood has potential benefits for the treatment of liver and brain diseases (Table 2).

## 4. Discussion

The umbilical cord is the most important channel between the fetus and the mother, and the umbilical cord is the lifeline of the fetus [1]. Current studies have found that animal umbilical venous blood and umbilical artery blood have differences in some physiological and biochemical indicators [22], which may be related to their different physiological functions. Umbilical venous blood is mainly responsible for the delivery of nutrients and oxygen, while umbilical arterial blood takes away the metabolic waste products and CO_2_ produced by the fetus [23]. Based on the phenotypic and functional differences between umbilical artery blood and umbilical venous blood, we speculate that there is a difference in miRNA expression between umbilical artery blood and umbilical venous blood. In addition, humoral miRNAs have been used in the study of disease markers for a long time, and many results have been obtained [24]. Corresponding researchers have also achieved animal and human disease intervention and treatment by inhibiting or overexpressing miRNA [25]. Although blood miRNAs have been used to assist in the diagnosis of human diseases, there are still few studies on cord blood miRNAs, and little is known about porcine cord blood miRNAs.

### 4.1. Characteristics of miRNA Expression in Swine Cord Venous Blood and Umbilical Artery Blood

High-abundance miRNAs may play a significant role in maintaining normal myocardial function [26]. Our data show that among the top 10 highly expressed miRNAs, miR-451-5p, miR-92a-3p, miR-486-5p, and miR-16-5p overlap in UVB and UAB. miR-451-5p and miR-486-5p are widely reported being involved in the formation and red subdivision of blood cells [27,28], and miR-92a-3p [29] and miR-16-5p [30] have also been reported to be involved in embryo and organ development. The expression of these four miRNAs accounted for more than 60.14% and 50.37% in UVB and UAB, respectively.

Functional enrichment analysis of miRNAs obtained by the two analytical methods shows that the signal pathways involved in UVB and UAB miRNAs have high similarities, mainly in the MAPK signaling pathway, endocytosis, focal adhesion, regulation of actin cytoskeleton, and pathways in cancer. Taken together, these results and previous reports have suggested that MAPK, endocytosis, focal adhesion, and regulation of actin cytoskeleton signaling pathways are of great significance for fetal development [31]. Both differential expression analysis methods show that UAB miRNAs have richer biological functions (more abundant pathways). This implies that UAB miRNA has a higher potential than UVB miRNA in reflecting the health of piglets, which is also consistent with the circulation of cord blood in the fetus [32].

### 4.2. Potential Application of miRNAs from Porcine Cord Venous Blood and Cord Arterial Blood

Since the discovery of cross-species spread of miRNAs, naturally occurring miRNAs have been used as potential drugs [8]. Because miRNA sequences are highly conserved among different species, and mature miRNAs have shorter sequences, they have low immunogenicity. Farm animals are easy to obtain in large quantities and stably, so related research based on farm animals has great application potential [33]. The traditional differential gene expression method uses differential expression multiples as the main analysis method [16]. This analysis method can quickly discover possible key genes and can be quickly verified by the current, widely popular RNA expression detection method. However, for the application research of animal-derived miRNAs, the higher the expression level of miRNAs, the easier it is to perform operations such as separation and purification, and the greater its application potential. Therefore, the analytical method of the expression difference percentage can take into account both the level of miRNA expression within the group and the analysis of the expression difference between groups.

Cord blood miRNA can be used to monitor the health of newborns [34] and can also be used to predict related metabolic diseases [35]. In addition, studies have reported that miRNAs secreted by umbilical cord blood mesenchymal stem cells are used to treat diseases [36]. RT-qPCR was performed on the miRNAs screened by the two analytical methods, and the results showed that only miR-19a-3p did not match the sequencing results, indicating that the use of the two analysis methods can further reduce false positives. Further analysis of the miRNAs of |log2Fold change| ≥ 2 and |UV-UA| ≥ 0.5 (it was higher than 90% miRNAs in UVB and 85% miRNAs in UAB) showed that miR-191-5p and miR-423-3p are highly expressed in UVB, and only miR-122-5p is highly expressed in UAB. The tissue expression profile showed that miR-423-3p expressed the highest in the placenta, while miR-122-5p expressed the lowest in the placenta, suggesting that miR-423-3p in umbilical cord blood may be derived from the placenta, while miR-122-5p in umbilical cord blood may be derived from a pig fetus. Relevant studies have shown that miR-122-5p may be involved in the migration and differentiation of fetal neurons [37], but miR-191-5p does not follow a similar rule. Therefore, miR-423-3p and miR-122-5p can be used as characteristic miRNAs of cord venous blood and cord arterial blood, respectively. Furthermore, we use MNDR [38] for disease analysis of miR-423-3p and miR-122-5p. It is found that the down-regulation of miR-423-3p is mainly involved in muscle-related diseases, while the down-regulation of miR-122-5p is related to liver- and brain-related diseases. These analyses suggest that UVB miRNAs have potential in the treatment of muscle-related diseases, while UAB miRNAs may be helpful in the treatment of liver- and brain-related diseases. When developing and utilizing umbilical cord blood, it is necessary to make targeted development and utilization based on its different characteristics to fully tap its application potential.

## 5. Conclusions

This study found that pig UVB and UAB have no significant differences in blood routine, but their miRNA expression profiles are different. Interestingly, use of the two analysis methods shows that the signal pathways involved in the high expression of miRNA in porcine UVB and UAB are mostly the same. The main difference is that the high expression of miRNA of UAB is more involved in cellular immunity and angiogenesis-related signal pathways. In addition, this study also found that miR-423-3p and miR-122-5p, as characteristic miRNAs of pig UVB and UAB, respectively, have potential application value for muscle-related diseases as well as liver- and brain-related diseases. These results will contribute to further understanding the role of miRNAs in the biological functions of cord blood and provide references for further research and application of porcine cord blood miRNA-related products.

## Figures and Tables

**Table 1:** Blood indexes of pig UVB and UAB.

Indexes	WBC (10^9^/L)	RBC (10^12^/L)	HGB (g/L)	HCT (%)	MCV (fL)
UV	19.43 ± 9.56	8.48 ± 1.27	112.67 ± 14.29	55.63 ± 9.65	65.53 ± 1.50
UA	35.7 ± 15.00	8.33 ± 0.98	115.33 ± 12.74	57.13 ± 9.58	68.40 ± 3.72

Note: white blood cell (WBC); red blood cell (RBC); hemoglobin (HGB); hematocrit (HCT); mean corpuscular volume (MCV).

**Table 2:** Types of diseases involved in characteristic miRNAs.

miRNA	Disease	Pattern	Tissue
miR-122-5p	AIDS dementia complex	Down	Brain
	Myasthenia gravis, autoimmune, experimental	Down	Blood
	Stroke, lacunar	Down	Blood
	Huntington’s disease	Down	Cell line
	Cardiovascular diseases	Down	Cell line
	Hepatitis C	Down	Liver
	Hepatitis B	Down	Hepatocytes
	Malignant glioma	Down	Cell line
	Amyotrophic lateral sclerosis	Down	Spinal cord
	Liver cancer	Down	Serum
	Cervical squamous cell carcinoma	Down	Tissue
	Hepatocellular carcinoma	Down	Serum
	Type 2 diabetes mellitus	Down	Cell line
	Fatty liver disease	Down	Cell line
	HCV	Down	Liver
miR-423-3p	Dermatomyositis	Down	Muscle
	Duchenne muscular dystrophy	Down	Muscle
	Temporal lobe epilepsy	Down	Brain
	Inclusion body myositis	Down	Muscle

All data are from NMDR (http://www.rna-society.org/mndr/home.html, accessed on 8 February 2021).

## Data Availability

Not applicable.

## Supplementary Materials

The following are available online at https://www.mdpi.com/article/10.3390/ani11061563/s1: Table S1: Sequences of PCR primers used in this study; Table S2: GO and KEGG analysis of differentially expressed miRNAs in pig UVB and UAB.
