# Supplementary material for: Expression Characteristics of microRNA in Pig Umbilical Venous Blood and Umbilical Arterial Blood
Source: Animals (Basel). 2021 May 27;11(6):1563. doi: 10.3390/ani11061563 (PMC8228062; doi:10.3390/ani11061563)
Supplement: Supplementary file 1 [file animals-11-01563-s001.zip › 2021.5.20-R2 Figure.pptx]

## Slide 1
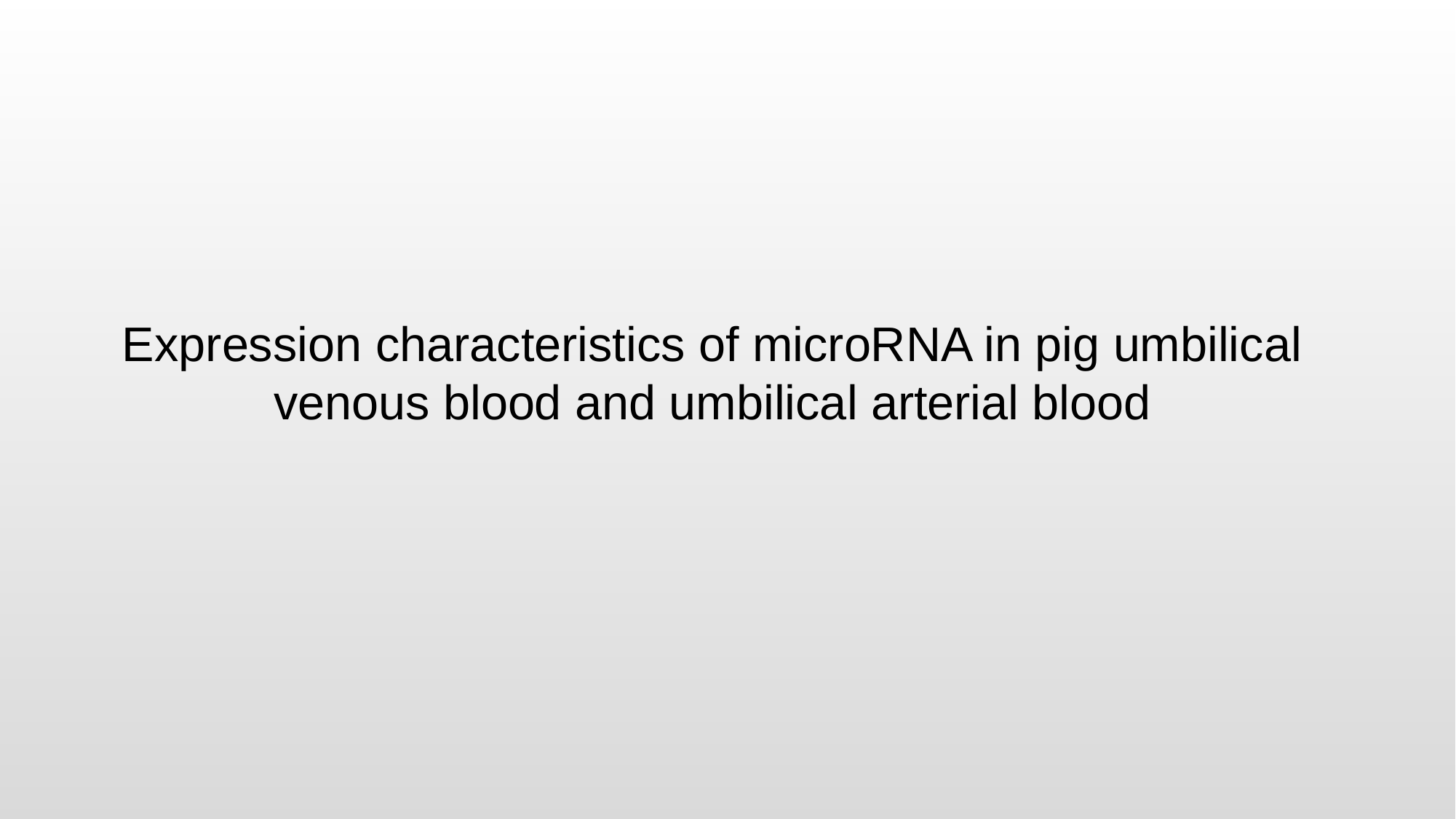

Expression characteristics of microRNA in pig umbilical venous blood and umbilical arterial blood

## Slide 2
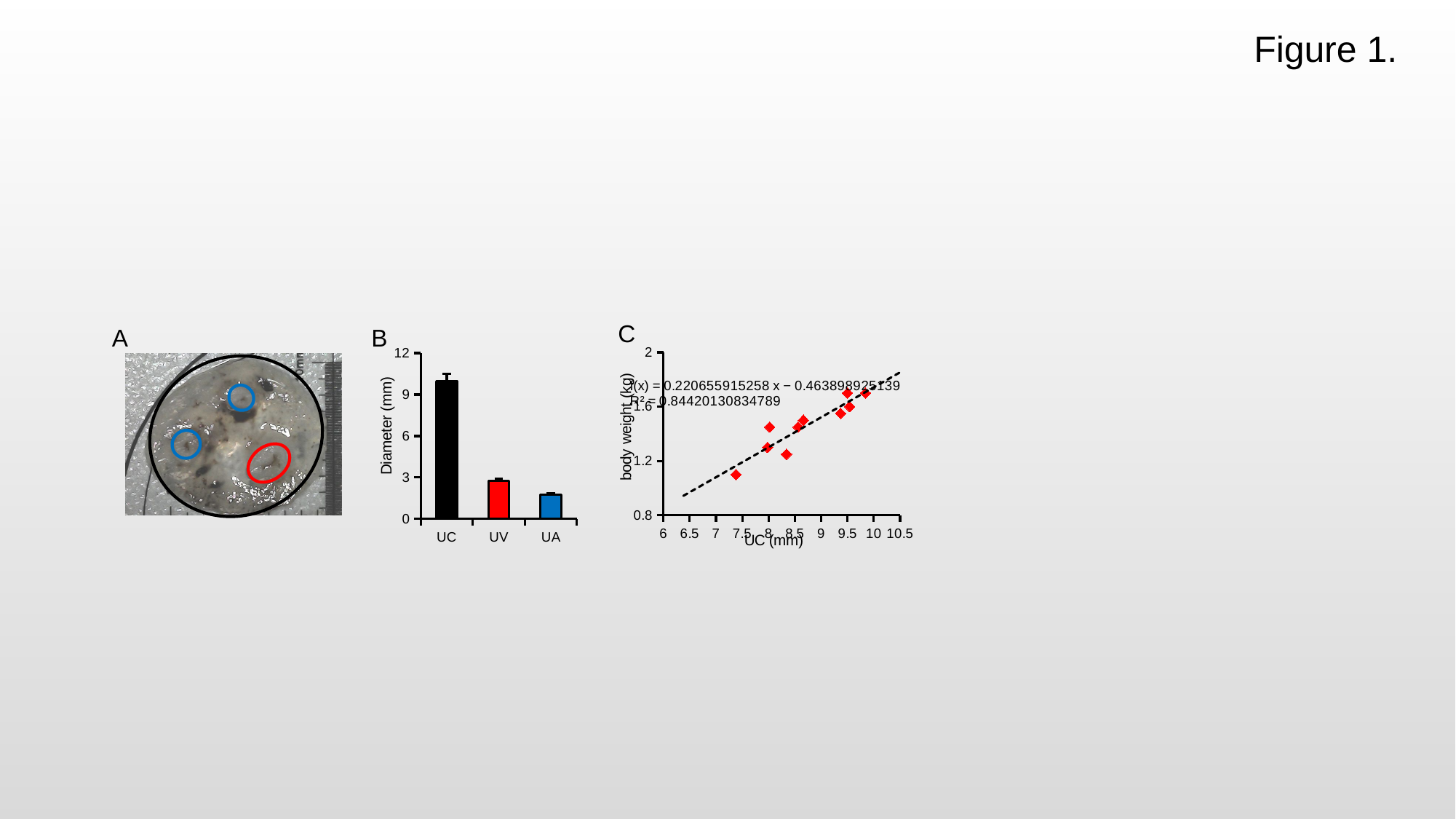

Figure 1.
C
A
B
### Chart
| Category | |
|---|---|
| UC | 9.942 |
| UV | 2.742 |
| UA | 1.766 |
### Chart
| Category | |
|---|---|

## Slide 3
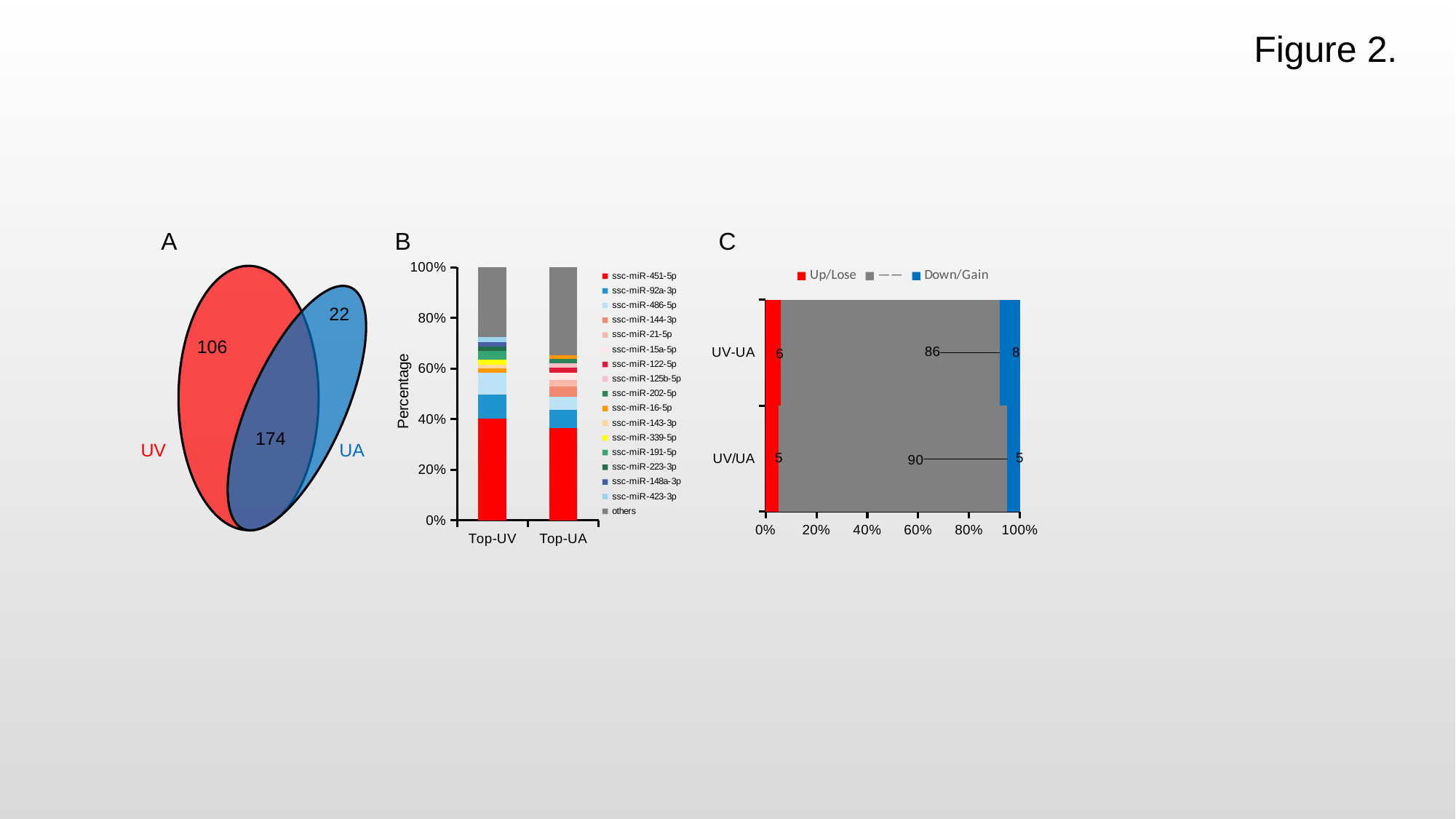

Figure 2.
A
B
C
### Chart
| Category | ssc-miR-451-5p | ssc-miR-92a-3p | ssc-miR-486-5p | ssc-miR-144-3p | ssc-miR-21-5p | ssc-miR-15a-5p | ssc-miR-122-5p | ssc-miR-125b-5p | ssc-miR-202-5p | ssc-miR-16-5p | ssc-miR-143-3p | ssc-miR-339-5p | ssc-miR-191-5p | ssc-miR-223-3p | ssc-miR-148a-3p | ssc-miR-423-3p | others |
|---|---|---|---|---|---|---|---|---|---|---|---|---|---|---|---|---|---|
| Top-UV | 40.2403841566143 | 9.47880363502577 | 8.60726262965763 | None | None | None | None | None | None | 1.80987894910391 | 1.51088312974404 | 1.77011886674223 | 3.65792757727504 | 1.6349345867125 | 1.81465015898731 | 1.94029201925024 | 27.534864290887 |
| Top-UA | 36.3613455965731 | 7.28738818193275 | 5.17071941539627 | 4.05190878165555 | 2.76678845911554 | 2.67607408340683 | 2.01083532820966 | 1.81428751417413 | 1.7084540758473 | 1.54970391835707 | None | None | None | None | None | None | 34.6024946453318 |
22
106
174
UV
UA
### Chart
| Category | Up/Lose | —— | Down/Gain |
|---|---|---|---|
| UV/UA | 5.0 | 90.0 | 5.0 |
| UV-UA | 6.0 | 86.0 | 8.0 |

## Slide 4
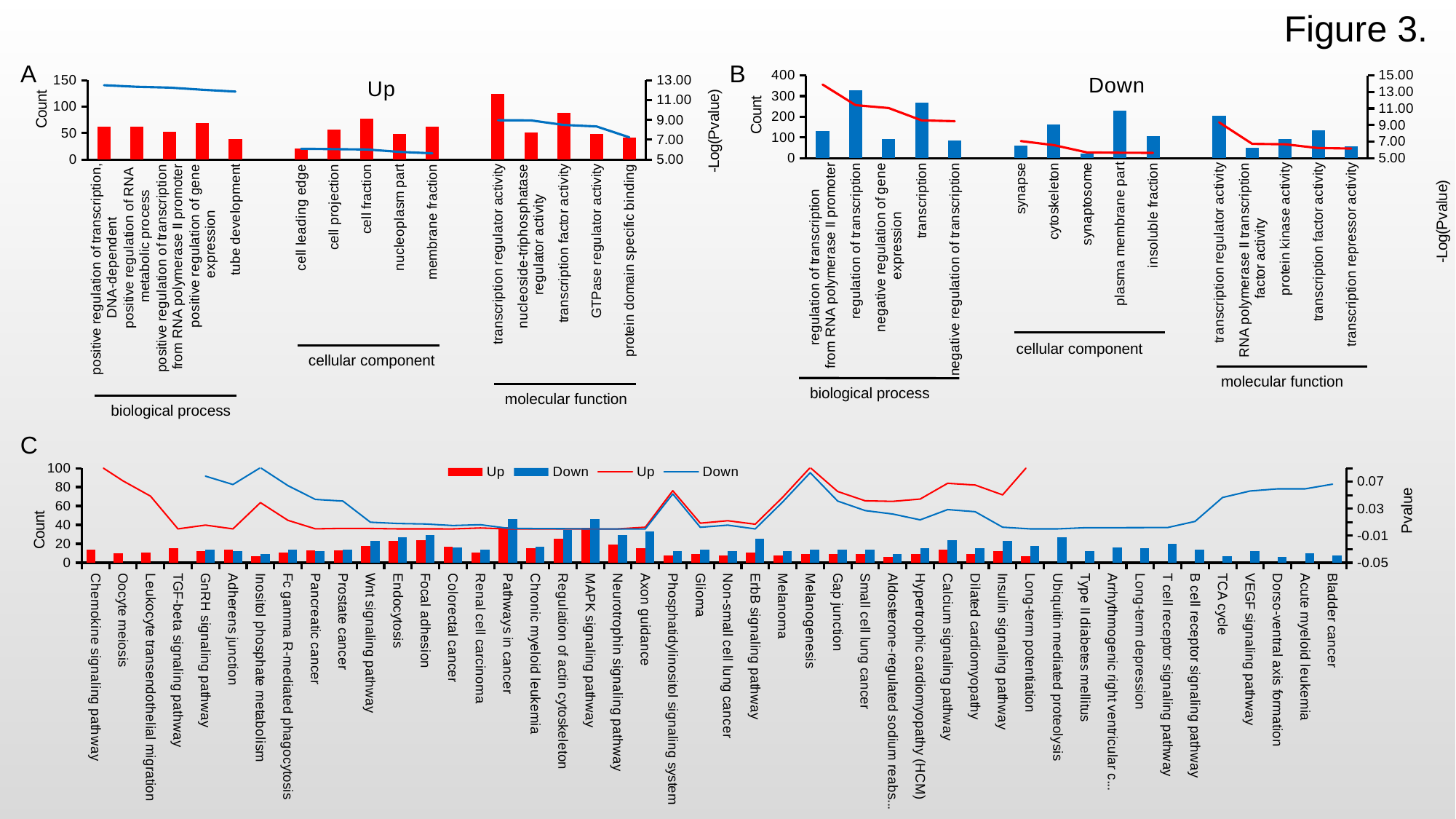

Figure 3.
A
### Chart: Up
| Category | Count | -Log(Pvalue) |
|---|---|---|
| positive regulation of transcription,
 DNA-dependent | 62.0 | 12.4982578669245 |
| positive regulation of RNA
metabolic process | 62.0 | 12.337115227812 |
| positive regulation of transcription
 from RNA polymerase II promoter | 53.0 | 12.2576127019348 |
| positive regulation of gene
 expression | 69.0 | 12.0409622410345 |
| tube development | 39.0 | 11.8577961569774 |
| | None | None |
| cell leading edge | 21.0 | 6.0894938039917 |
| cell projection | 57.0 | 6.0526899155285 |
| cell fraction | 78.0 | 6.01626330543905 |
| nucleoplasm part | 48.0 | 5.77498294963477 |
| membrane fraction | 62.0 | 5.6317067131755 |
| | None | None |
| transcription regulator activity | 124.0 | 8.96703797202176 |
| nucleoside-triphosphatase
 regulator activity | 51.0 | 8.95096547569122 |
| transcription factor activity | 89.0 | 8.49389763969001 |
| GTPase regulator activity | 49.0 | 8.34147264171139 |
| protein domain specific binding | 41.0 | 7.24023289920234 |cellular component
molecular function
biological process
B
### Chart: Down
| Category | Count | -Log(Pvalue) |
|---|---|---|
| regulation of transcription
from RNA polymerase II promoter | 130.0 | 13.894480369584 |
| regulation of transcription | 328.0 | 11.4021054641896 |
| negative regulation of gene
 expression | 94.0 | 11.0567983919315 |
| transcription | 268.0 | 9.56306734484123 |
| negative regulation of transcription | 84.0 | 9.46752847697568 |
| | None | None |
| synapse | 59.0 | 7.06466908565277 |
| cytoskeleton | 161.0 | 6.5729588538471 |
| synaptosome | 22.0 | 5.69410158219926 |
| plasma membrane part | 231.0 | 5.65349666251167 |
| insoluble fraction | 105.0 | 5.6366913599644 |
| | None | None |
| transcription regulator activity | 206.0 | 9.32653750612381 |
| RNA polymerase II transcription
 factor activity | 49.0 | 6.72902506302812 |
| protein kinase activity | 94.0 | 6.67676264645259 |
| transcription factor activity | 134.0 | 6.21720610399755 |
| transcription repressor activity | 57.0 | 6.16044066311247 |cellular component
molecular function
biological process
C
### Chart
| Category | Up | Down | Up | Down |
|---|---|---|---|---|
| Chemokine signaling pathway | 14.0 | None | 0.0977018110279596 | None |
| Oocyte meiosis | 10.0 | None | 0.0711758573356294 | None |
| Leukocyte transendothelial migration | 11.0 | None | 0.048558548243588 | None |
| TGF-beta signaling pathway | 15.0 | None | 4.19333697862495e-05 | None |
| GnRH signaling pathway | 12.0 | 14.0 | 0.00569915581841363 | 0.0781772494067588 |
| Adherens junction | 14.0 | 12.0 | 4.70982618536778e-05 | 0.065714754056004 |
| Inositol phosphate metabolism | 7.0 | 9.0 | 0.0389749452388636 | 0.0907719080450901 |
| Fc gamma R-mediated phagocytosis | 11.0 | 14.0 | 0.0127457722725681 | 0.0640891194232885 |
| Pancreatic cancer | 13.0 | 12.0 | 0.000105295819927559 | 0.0436825175385276 |
| Prostate cancer | 13.0 | 14.0 | 0.000802105581365825 | 0.0412343901938336 |
| Wnt signaling pathway | 18.0 | 23.0 | 0.00061230451821999 | 0.00997988566555053 |
| Endocytosis | 23.0 | 27.0 | 3.8547411743017e-05 | 0.00798268965170595 |
| Focal adhesion | 24.0 | 29.0 | 5.17162336135085e-05 | 0.00732154213425469 |
| Colorectal cancer | 17.0 | 16.0 | 1.18132201951508e-06 | 0.0050160441008377 |
| Renal cell carcinoma | 11.0 | 14.0 | 0.00138922234627693 | 0.00622621954100816 |
| Pathways in cancer | 38.0 | 46.0 | 3.08182827143783e-07 | 0.00103002896803278 |
| Chronic myeloid leukemia | 15.0 | 17.0 | 7.19234942744366e-06 | 0.000533265601685255 |
| Regulation of actin cytoskeleton | 25.0 | 35.0 | 5.31066303932575e-05 | 0.000340202771352564 |
| MAPK signaling pathway | 37.0 | 46.0 | 4.23638425496354e-09 | 6.89873386505441e-06 |
| Neurotrophin signaling pathway | 19.0 | 29.0 | 1.46469975435541e-05 | 1.45270180344464e-06 |
| Axon guidance | 15.0 | 33.0 | 0.00261106585384576 | 2.35387821125529e-08 |
| Phosphatidylinositol signaling system | 8.0 | 12.0 | 0.0565949830357379 | 0.051776720514667 |
| Glioma | 9.0 | 14.0 | 0.00852744583215444 | 0.00239693978286597 |
| Non-small cell lung cancer | 8.0 | 12.0 | 0.0121712996129929 | 0.00562677265859278 |
| ErbB signaling pathway | 11.0 | 25.0 | 0.00698831670009184 | 1.66270790751718e-07 |
| Melanoma | 8.0 | 12.0 | 0.0469842932343651 | 0.0399815589989126 |
| Melanogenesis | 9.0 | 14.0 | 0.0909005639506072 | 0.0832824864244359 |
| Gap junction | 9.0 | 14.0 | 0.0554434598031099 | 0.0412343901938336 |
| Small cell lung cancer | 9.0 | 14.0 | 0.0416661294708457 | 0.0271617955901716 |
| Aldosterone-regulated sodium reabsorption | 6.0 | 9.0 | 0.0408452048859606 | 0.0220743012722528 |
| Hypertrophic cardiomyopathy (HCM) | 9.0 | 15.0 | 0.0442173593903324 | 0.0134141377943273 |
| Calcium signaling pathway | 14.0 | 24.0 | 0.067578341947999 | 0.0286456623635647 |
| Dilated cardiomyopathy | 9.0 | 15.0 | 0.0649601732407598 | 0.0255534178508997 |
| Insulin signaling pathway | 12.0 | 23.0 | 0.0503983051682134 | 0.00255144652596259 |
| Long-term potentiation | 7.0 | 18.0 | 0.0970913196611075 | 4.44014772839482e-05 |
| Ubiquitin mediated proteolysis | None | 27.0 | None | 8.90580417481523e-05 |
| Type II diabetes mellitus | None | 12.0 | None | 0.00177765724868319 |
| Arrhythmogenic right ventricular cardiomyopathy (ARVC) | None | 16.0 | None | 0.00182857262764698 |
| Long-term depression | None | 15.0 | None | 0.0019560104062625 |
| T cell receptor signaling pathway | None | 20.0 | None | 0.00201241196273861 |
| B cell receptor signaling pathway | None | 14.0 | None | 0.0111727352600398 |
| TCA cycle | None | 7.0 | None | 0.0465484808887752 |
| VEGF signaling pathway | None | 12.0 | None | 0.0561792823518301 |
| Dorso-ventral axis formation | None | 6.0 | None | 0.0593352177236669 |
| Acute myeloid leukemia | None | 10.0 | None | 0.0593913528405978 |
| Bladder cancer | None | 8.0 | None | 0.0662640799034022 |

## Slide 5
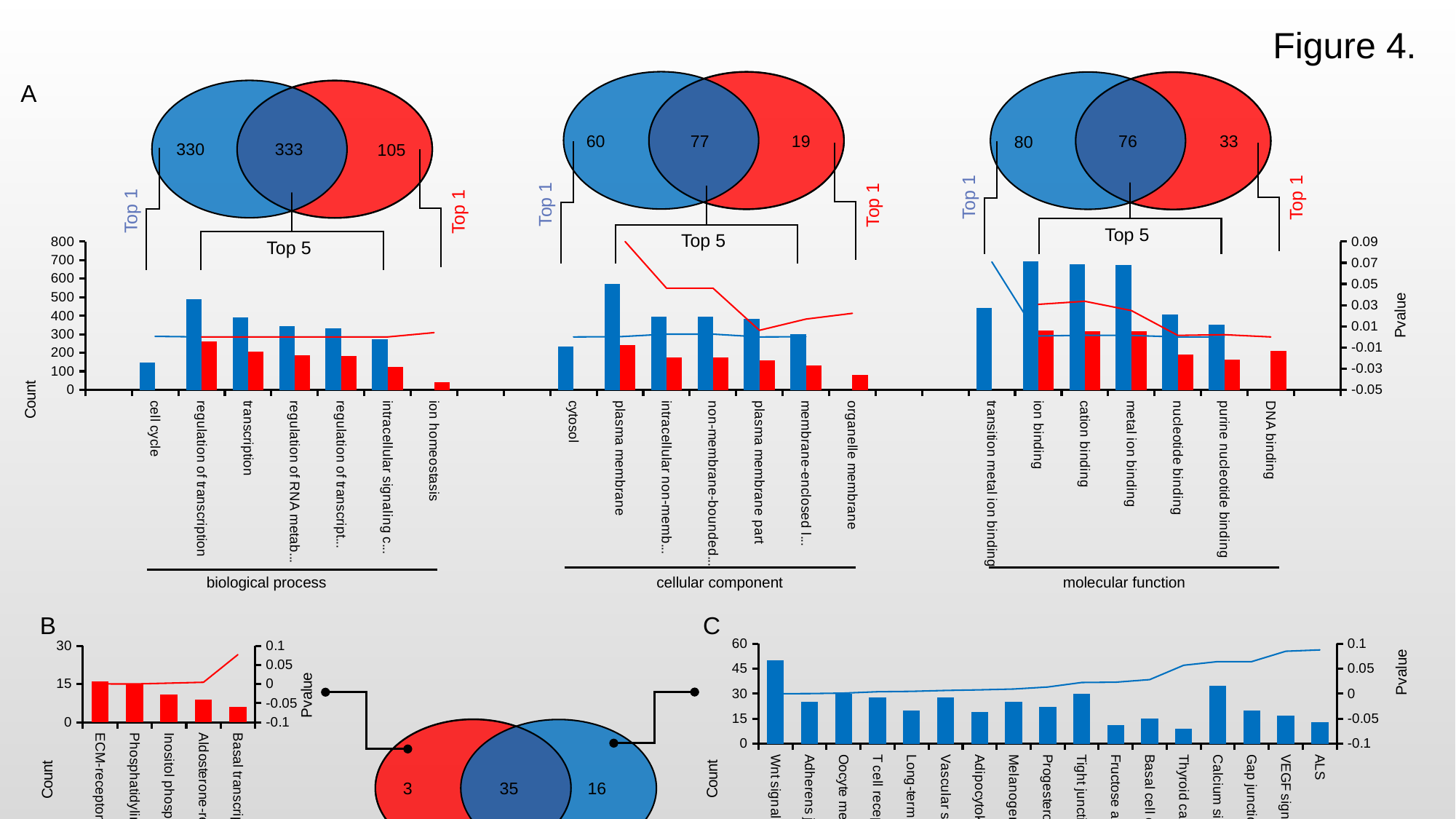

Figure 4.
77
19
60
76
33
80
333
105
330
Top 1
Top 1
Top 5
Top 1
Top 1
Top 5
Top 1
Top 1
Top 5
### Chart
| Category | gain | lose | gain | lose |
|---|---|---|---|---|
| | None | None | None | None |
| cell cycle | 148.0 | None | 0.000528432600503266 | None |
| regulation of transcription | 488.0 | 260.0 | 2.26414960541966e-10 | 2.17022154476945e-12 |
| transcription | 389.0 | 206.0 | 1.54775836417648e-07 | 6.08127394259993e-09 |
| regulation of RNA metabolic process | 343.0 | 188.0 | 1.15582328975418e-07 | 4.37568959225206e-10 |
| regulation of transcription, DNA-dependent | 332.0 | 182.0 | 5.52372072777374e-07 | 2.11781590309982e-09 |
| intracellular signaling cascade | 274.0 | 126.0 | 1.45609414473506e-12 | 3.80668071972197e-06 |
| ion homeostasis | None | 43.0 | None | 0.00407254540505042 |
| | None | None | None | None |
| | None | None | None | None |
| cytosol | 233.0 | None | 8.06229549031597e-06 | None |
| plasma membrane | 572.0 | 242.0 | 0.000246326087287107 | 0.0955209321704515 |
| intracellular non-membrane-bounded organelle | 395.0 | 174.0 | 0.00260463786362169 | 0.0460695131097294 |
| non-membrane-bounded organelle | 395.0 | 174.0 | 0.00260463786362169 | 0.0460695131097294 |
| plasma membrane part | 385.0 | 158.0 | 3.32735529515851e-09 | 0.00623755051647401 |
| membrane-enclosed lumen | 301.0 | 132.0 | 0.00017266942871188 | 0.0168649359976186 |
| organelle membrane | None | 82.0 | None | 0.0222637945974976 |
| | None | None | None | None |
| | None | None | None | None |
| transition metal ion binding | 441.0 | None | 0.0708529460623431 | None |
| ion binding | 692.0 | 322.0 | 0.000903343496345251 | 0.0307419416487839 |
| cation binding | 679.0 | 317.0 | 0.00158816486358214 | 0.0336440371320949 |
| metal ion binding | 674.0 | 316.0 | 0.00143266424846488 | 0.0249350043808294 |
| nucleotide binding | 408.0 | 191.0 | 1.81306518459317e-06 | 0.00147237412572909 |
| purine nucleotide binding | 351.0 | 165.0 | 6.23243884307753e-06 | 0.0021177505288062 |
| DNA binding | None | 210.0 | None | 2.68205517019379e-05 |
| | None | None | None | None |A
biological process
cellular component
molecular function
B
C
### Chart
| Category | | |
|---|---|---|
| Wnt signaling pathway | 50.0 | 3.14358599115886e-08 |
| Adherens junction | 25.0 | 0.000220571505588234 |
| Oocyte meiosis | 30.0 | 0.00117499284126352 |
| T cell receptor signaling pathway | 28.0 | 0.00388922635264882 |
| Long-term depression | 20.0 | 0.0047604932016433 |
| Vascular smooth muscle contraction | 28.0 | 0.00659421921706174 |
| Adipocytokine signaling pathway | 19.0 | 0.0078081995434779 |
| Melanogenesis | 25.0 | 0.00940087215482667 |
| Progesterone-mediated oocyte maturation | 22.0 | 0.0134208866077274 |
| Tight junction | 30.0 | 0.0224321690186314 |
| Fructose and mannose metabolism | 11.0 | 0.0230334241994415 |
| Basal cell carcinoma | 15.0 | 0.0281594486730313 |
| Thyroid cancer | 9.0 | 0.0570144505014032 |
| Calcium signaling pathway | 35.0 | 0.0642333967402528 |
| Gap junction | 20.0 | 0.064247761002462 |
| VEGF signaling pathway | 17.0 | 0.0850316841625395 |
| ALS | 13.0 | 0.0876571800171792 |
35
3
16
D
### Chart
| Category | lose | gain | lose | gain |
|---|---|---|---|---|
| Fc epsilon RI signaling pathway | 11.0 | 18.0 | 0.0308502339488168 | 0.0656178174176978 |
| Glioma | 13.0 | 17.0 | 0.000687667704065338 | 0.0201582903604831 |
| Dilated cardiomyopathy | 16.0 | 23.0 | 0.000867114762324724 | 0.0147508392093897 |
| Fc gamma R-mediated phagocytosis | 14.0 | 23.0 | 0.00885573247026193 | 0.0211730497036097 |
| Non-small cell lung cancer | 11.0 | 16.0 | 0.00237530310069203 | 0.0105789796367988 |
| HCM | 15.0 | 23.0 | 0.00115662141594422 | 0.00563686781384608 |
| Small cell lung cancer | 15.0 | 23.0 | 0.00102592565649725 | 0.00484002134760736 |
| Dorso-ventral axis formation | 6.0 | 9.0 | 0.0214313785286866 | 0.0246581243563535 |
| ErbB signaling pathway | 16.0 | 25.0 | 0.000471008662872063 | 0.00156393029536383 |
| Prostate cancer | 18.0 | 27.0 | 5.29282639553229e-05 | 0.000388114280520252 |
| Regulation of actin cytoskeleton | 35.0 | 55.0 | 1.0583853773129e-06 | 4.59143788357154e-05 |
| Focal adhesion | 34.0 | 53.0 | 6.41222167388526e-07 | 2.6861008854538e-05 |
| Pathways in cancer | 45.0 | 86.0 | 2.84272678622343e-06 | 5.96116976549625e-08 |
| Renal cell carcinoma | 14.0 | 24.0 | 0.000535437711199001 | 0.000122372594204453 |
| Melanoma | 13.0 | 22.0 | 0.00204846456823458 | 0.00115716839804642 |
| Acute myeloid leukemia | 11.0 | 19.0 | 0.00409566404699703 | 0.00139692572555382 |
| Neurotrophin signaling pathway | 18.0 | 41.0 | 0.00289028515388917 | 7.1315380275826e-07 |
| Insulin signaling pathway | 19.0 | 46.0 | 0.00300003856587946 | 4.72346076612838e-08 |
| Chronic myeloid leukemia | 13.0 | 26.0 | 0.00329567484363312 | 4.77422370360111e-05 |
| TGF-beta signaling pathway | 14.0 | 30.0 | 0.00417839548591644 | 1.19955912447077e-05 |
| mTOR signaling pathway | 10.0 | 18.0 | 0.00618791722372963 | 0.000985095608171271 |
| Pancreatic cancer | 12.0 | 22.0 | 0.00689165412356705 | 0.0014095316884605 |
| Colorectal cancer | 13.0 | 28.0 | 0.00836490489785589 | 4.97389922596597e-05 |
| Axon guidance | 17.0 | 41.0 | 0.0100486705154308 | 2.22683826816188e-06 |
| p53 signaling pathway | 10.0 | 18.0 | 0.0329793031184648 | 0.0197179956987866 |
| Ubiquitin mediated proteolysis | 17.0 | 37.0 | 0.0172911529019969 | 0.000336033096531323 |
| ARVC | 11.0 | 23.0 | 0.0262685904979826 | 0.00122163926181723 |
| GnRH signaling pathway | 12.0 | 23.0 | 0.0561056945857496 | 0.0295766466835756 |
| MAPK signaling pathway | 27.0 | 79.0 | 0.0271467791269961 | 6.48790415287703e-10 |
| Type II diabetes mellitus | 8.0 | 17.0 | 0.0325357083248069 | 0.000840535262136378 |
| Endocytosis | 20.0 | 50.0 | 0.0326567005809086 | 2.01185958422432e-05 |
| Endometrial cancer | 8.0 | 15.0 | 0.0524142631864069 | 0.0175387671208755 |
| Cell cycle | 14.0 | 29.0 | 0.0664623328213315 | 0.0158654673384246 |
| Long-term potentiation | 9.0 | 24.0 | 0.076559700685123 | 7.37639919091462e-05 |
| Notch signaling pathway | 7.0 | 16.0 | 0.0859681944076656 | 0.00252466170260297 |
### Chart
| Category | | |
|---|---|---|
| ECM-receptor interaction | 16.0 | 0.000317649191268648 |
| Phosphatidylinositol signaling system | 15.0 | 0.000269033641956773 |
| Inositol phosphate metabolism | 11.0 | 0.00237530310069203 |
| Aldosterone-regulated sodium reabsorption | 9.0 | 0.00457613010420601 |
| Basal transcription factors | 6.0 | 0.0768905824537449 |

## Slide 6
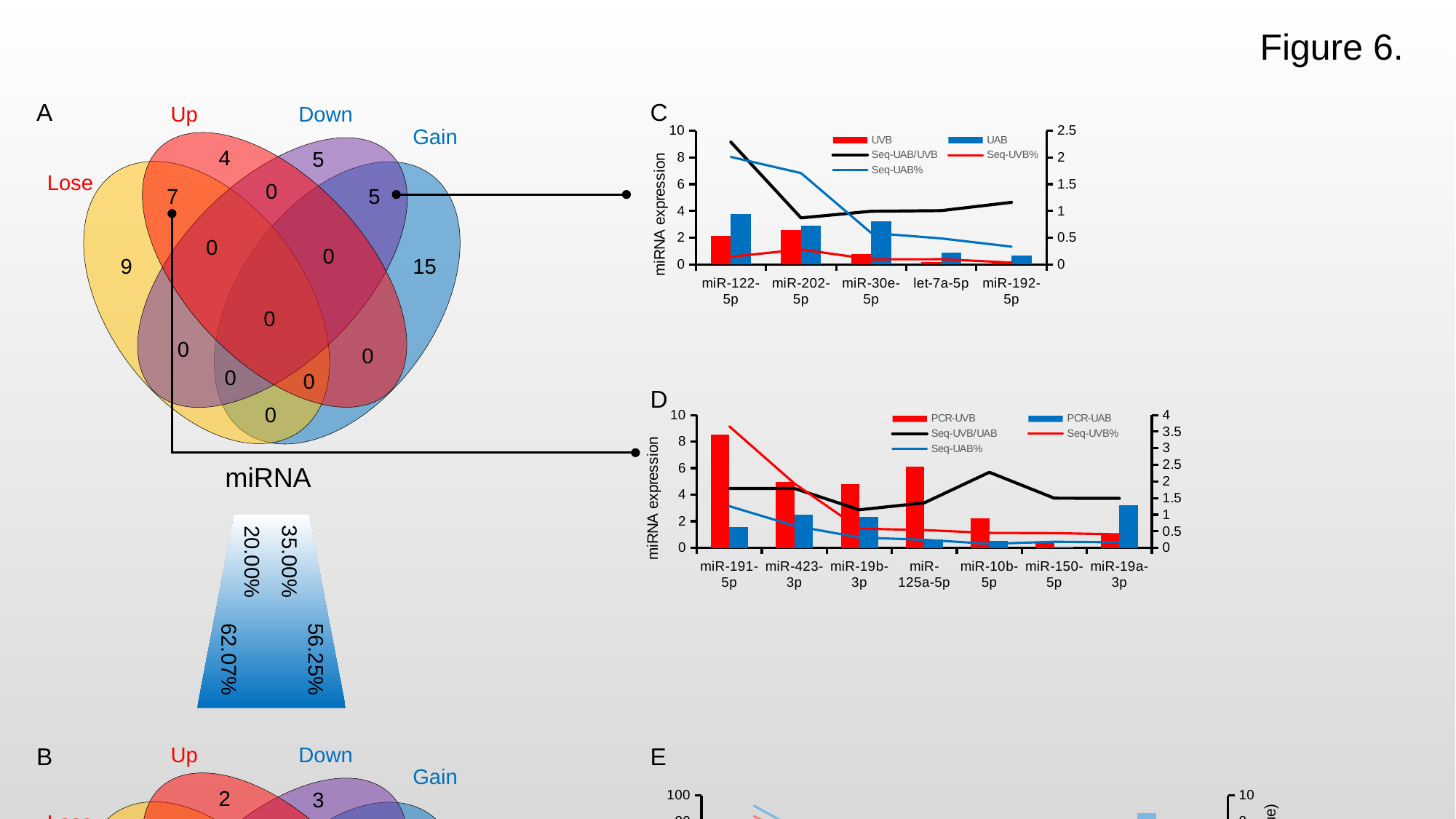

Figure 6.
A
C
Up
Down
Gain
Lose
5
15
4
0
7
5
0
0
9
0
0
0
0
0
0
miRNA
### Chart
| Category | UVB | UAB | Seq-UAB/UVB | Seq-UVB% | Seq-UAB% |
|---|---|---|---|---|---|
| miR-122-5p | 2.14507582021142 | 3.75980354782162 | 9.15503075385468 | 0.143136296502067 | 2.01083532820966 |
| miR-202-5p | 2.5531368402966 | 2.87545580301484 | 3.48476264596913 | 0.279910979826264 | 1.7084540758473 |
| miR-30e-5p | 0.76646414874471 | 3.21887307218709 | 3.97755736358092 | 0.0954241976680446 | 0.58964344210659 |
| let-7a-5p | 0.20043705043613 | 0.874250873653699 | 4.02683683534209 | 0.0974387085077032 | 0.486329847549454 |
| miR-192-5p | 0.22214668793493 | 0.66327770480931 | 4.64635019462551 | 0.0349888724782831 | 0.332619377598589 |D
### Chart
| Category | PCR-UVB | PCR-UAB | Seq-UVB/UAB | Seq-UVB% | Seq-UAB% |
|---|---|---|---|---|---|
| miR-191-5p | 8.52181582060837 | 1.55914933048953 | 4.47300174536147 | 3.65792757727504 | 1.25488219730377 |
| miR-423-3p | 4.9678215581219 | 2.52082118064376 | 4.47565372268086 | 1.94029201925024 | 0.665238755197179 |
| miR-19b-3p | 4.80997853430081 | 2.3047825230448 | 2.86613634635971 | 0.578906799186137 | 0.309940783671414 |
| miR-125a-5p | 6.10067580276283 | 0.616167081929738 | 3.38975740963698 | 0.534375506941051 | 0.241905001889883 |
| miR-10b-5p | 2.23053727266046 | 0.511177222319378 | 5.68994993760493 | 0.44849372903981 | 0.120952500944942 |
| miR-150-5p | 0.331573116512869 | 0.0960287723827098 | 3.73949428523445 | 0.442132115861941 | 0.181428751417413 |
| miR-19a-3p | 1.0553364506961 | 3.23523371539548 | 3.72726572315062 | 0.403962436794722 | 0.166309688799295 |
56.25%
35.00%
20.00%
62.07%
B
Up
Down
Gain
Lose
3
8
2
0
0
3
3
5
2
23
0
1
5
1
6
KEGG Pathway
E
### Chart
| Category | Gain | Down | Lose | Up | Gain | Down | Lose | Up |
|---|---|---|---|---|---|---|---|---|
| MAPK signaling pathway | 79.0 | 46.0 | 27.0 | 37.0 | 9.18789557466991 | 5.16123060866519 | 1.56628169058162 | 8.37300465473977 |
| Endocytosis | 50.0 | 27.0 | 20.0 | 23.0 | 4.69640233371779 | 2.09785075475395 | 1.48602769565229 | 4.41400477723591 |
| Focal adhesion | 53.0 | 29.0 | 34.0 | 24.0 | 4.57087767998708 | 2.13539743398825 | 6.19299147226907 | 4.2863731114102 |
| Regulation of actin cytoskeleton | 55.0 | 35.0 | 35.0 | 25.0 | 4.33805128676996 | 3.46826215286859 | 5.97535616900592 | 4.27485125361833 |
| Pathways in cancer | 86.0 | 46.0 | 45.0 | 38.0 | 7.22466850991547 | 2.98715056123669 | 5.54626487824038 | 6.511191565113 |Top 5

## Slide 7
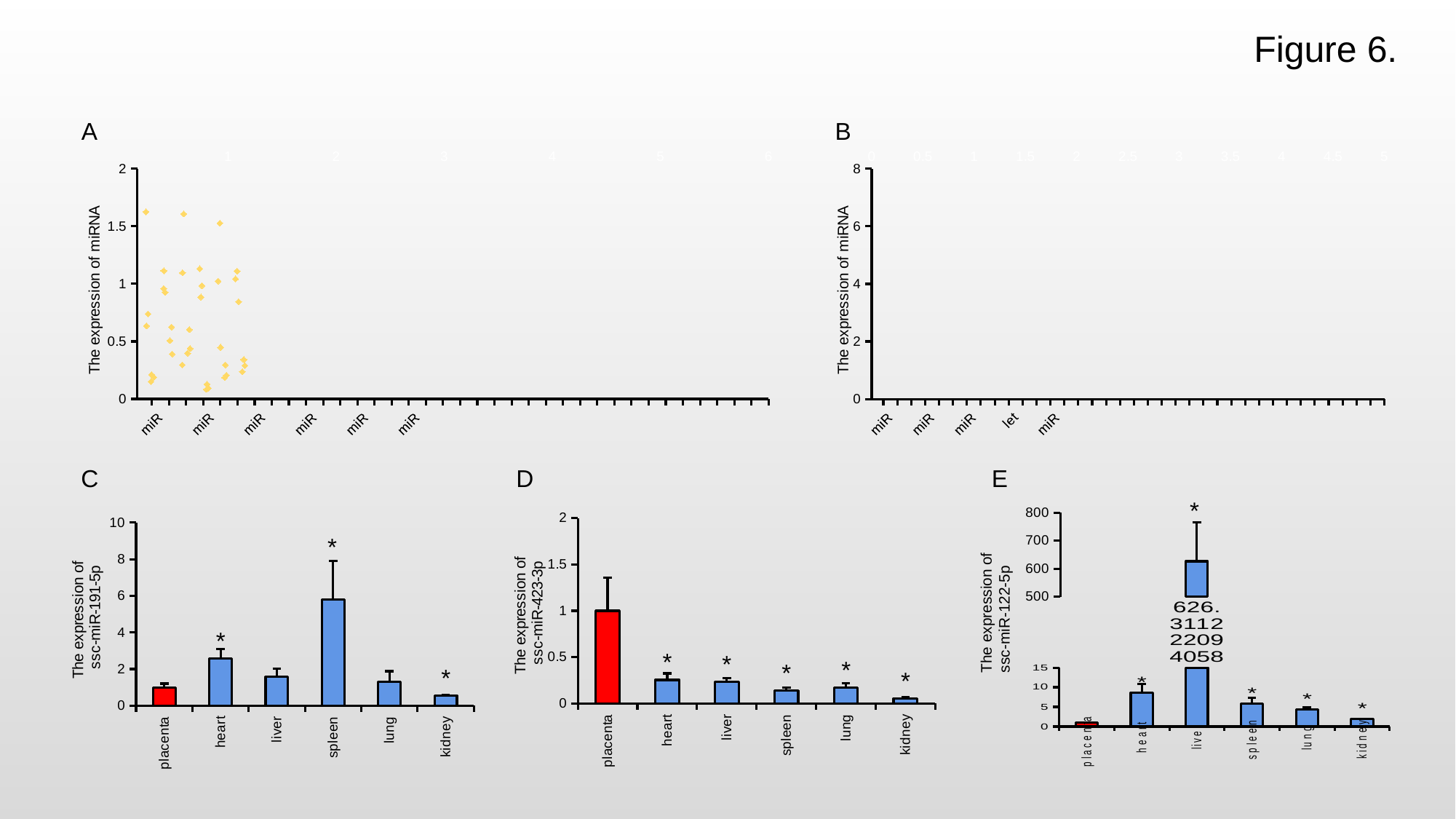

Figure 6.
A
B
### Chart
| Category | | |
|---|---|---|
| miR-191-5p | 1.0 | 0.635168561110381 |
| | 0.182959754506666 | 0.738986730953583 |
| | None | 1.62584470793604 |
| miR-423-3p | 1.0 | 0.188210455353803 |
| | 0.507429896817142 | 0.150091967913537 |
| | None | 0.21057684025266 |
| miR-19b-3p | 1.0 | 0.959740905010361 |
| | 0.4791669040951 | 0.926998627771416 |
| | None | 1.11326046721822 |
| miR-125a-5p | 1.0 | 0.507429896817142 |
| | 0.100999807537829 | 0.625016506663767 |
| | None | 0.389843286970516 |
| miR-10b-5p | 1.0 | 1.09622891565417 |
| | 0.229172239614572 | 0.295522579516132 |
| | None | 1.60824850482969 |
| miR-150-5p | 1.0 | 0.602297883698161 |
| | 0.289615676302825 | 0.437558887222113 |
### Chart
| Category | | |
|---|---|---|
| miR-122-5p | 1.0 | 1.13471663984034 |
| | 1.75276021126892 | 0.946998020777492 |
| | None | 0.91828533938217 |
| miR-202-5p | 1.0 | 1.79937861004894 |
| | 1.12624429589164 | 2.07917100511755 |
| | None | 1.37973101864027 |
| miR-30e-5p | 1.0 | 0.566170578930033 |
| | 4.19963944492231 | 1.58042074268385 |
| | None | 0.853408678386119 |
| let-7a-5p | 1.0 | 1.31522414237335 |
| | 4.36172290378161 | 1.48947993257539 |
| | None | 0.57402881272617 |
| miR-192-5p | 1.0 | 1.03906746828808 |
| | 2.98576454582835 | 0.868314351338934 |C
D
E
### Chart
| Category | ssc-miR-423-3p |
|---|---|
| placenta | 1.0 |
| heart | 0.253724573505689 |
| liver | 0.234557736313646 |
| spleen | 0.138658936488735 |
| lung | 0.169507291203668 |
| kidney | 0.054777868135628 |
### Chart
| Category | ssc-miR-191-5p |
|---|---|
| placenta | 1.0 |
| heart | 2.57186496036626 |
| liver | 1.56666707579812 |
| spleen | 5.78119528654605 |
| lung | 1.28702967360458 |
| kidney | 0.548411407151506 |
### Chart
| Category | ssc-miR-122-5p |
|---|---|
| placenta | 1.0 |
| heart | 8.62256473486759 |
| liver | 626.311222094058 |
| spleen | 5.770303201533 |
| lung | 4.30369180325981 |
| kidney | 1.88799405433447 |
### Chart
| Category | ssc-miR-122-5p |
|---|---|
| placenta | 1.0 |
| heart | 8.62256473486759 |
| liver | 626.311222094058 |
| spleen | 5.770303201533 |
| lung | 4.30369180325981 |
| kidney | 1.88799405433447 |The expression of
ssc-miR-122-5p
